# Cleft Palate, Moderate Lung Developmental Retardation and Early Postnatal Lethality in Mice Deficient in the Kir7.1 Inwardly Rectifying K^+^ Channel

**DOI:** 10.1371/journal.pone.0139284

**Published:** 2015-09-24

**Authors:** Sandra Villanueva, Johanna Burgos, Karen I. López-Cayuqueo, Ka-Man Venus Lai, David M. Valenzuela, L. Pablo Cid, Francisco V. Sepúlveda

**Affiliations:** 1 Centro de Estudios Científicos (CECs), Valdivia, Chile; 2 Doctorado en Ciencias Veterinarias de la Universidad Austral de Chile, Valdivia, Chile; 3 Regeneron Pharmaceuticals, Inc., Tarrytown, New York, United States of America; Xuzhou Medical College, CHINA

## Abstract

Kir7.1 is an inwardly rectifying K^+^ channel of the Kir superfamily encoded by the *kcnj13* gene. Kir7.1 is present in epithelial tissues where it colocalizes with the Na^+^/K^+^-pump probably serving to recycle K^+^ taken up by the pump. Human mutations affecting Kir7.1 are associated with retinal degeneration diseases. We generated a mouse lacking Kir7.1 by ablation of the *Kcnj13* gene. Homozygous mutant null mice die hours after birth and show cleft palate and moderate retardation in lung development. Kir7.1 is expressed in the epithelium covering the palatal processes at the time at which palate sealing takes place and our results suggest it might play an essential role in late palatogenesis. Our work also reveals a second unexpected role in the development and the physiology of the respiratory system, where Kir7.1 is expressed in epithelial cells all along the respiratory tree.

## Introduction

Kir7.1 encoded by the *Kcnj13* gene is a K^+^ channel belonging to a group of membrane proteins described as K^+^-transport type channels of the inwardly-rectifying Kir family. Kir7.1 was first described in 1998 and it presents marked differences in sequence as well as functional properties when compared with those of other members of the Kir channel superfamily [1–3]. Also, Kir 7.1 channels are rather independent of voltage and K^+^ concentration and more akin to leak-type K^+^ channels, which is unusual for a Kir family channel whose other members present strong inward rectification properties, that is to say they allow mainly currents associated with K^+^ influx into cells [4].

Kir7.1 is present in epithelial tissues and shows a remarkable colocalization with the Na^+^/K^+^-pump. Kir7.1 is present at the basolateral membrane of intestinal epithelial cells, thyroid follicular cells and epithelial cells of proximal and distal convoluted tubule [5–7]. These epithelia express the Na^+^/K^+^-pump at the same location, which is the most usual in polarised cell layers. Interestingly Kir7.1 is expressed at the apical membrane of retinal pigmented epithelium (RPE) and in the choroid plexus, epithelia where exceptionally the pump is also expressed apically [5,8–10]. This colocalization with the Na^+^/K^+^-pump suggests that Kir7.1 could serve the role of K^+^ recycling needed to keep up with high rates of epithelial to ion transport [11]. An additional recently proposed function of Kir7.1 is in the control of excitability of uterine smooth muscle and in the regulation of the transition from quiescence to contractions in the pregnant uterus [12]. Also in a role in excitability regulation, Kir 7.1 has been proposed to play an important role in melanocortin-mediated regulation of energy homeostasis within the paraventricular nucleus where coupling of melanocortin receptor 4 to Kir7.1 could account for aspects of the control of food intake [13].

Mutations in human *KCNJ13* gene are associated with retinal diseases snowflake vitreoretinal degeneration (SVD) [14] and Leber congenital amaurosis [15,16]. The mutations have been shown to lead to trafficking or functional defects [11]. Recently, Zhong et al. [17] have generated mosaic *Kcnj13* expression in the RPE and shown that those photoreceptors apposing RPE cells lacking, but not those expressing Kir7.1, degenerate. This suggests a role of Kir7.1 in the ionic regulation of the confined space between RPE and photoreceptors possibly in relation to lactate transport, perhaps in the way it has been proposed to explain a similar phenotype in mice deficient in the ClC-2 Cl^-^ channel [18].

In order to study the role of Kir7.1 in epithelial transport we generated a Kir 7.1 deficient mouse by ablating the *Kcnj13* gene. Homozygous null mutant mice die hours after birth and show cleft palate and moderate retardation in lung development. Kir7.1 is expressed in the epithelium covering the palatal processes at the time at which palate sealing takes place and our results suggest this channel has an essential role in late palatogenesis. Our work also reveals in addition a second unexpected role in the development and physiology of the respiratory system.

## Materials and Methods

### Mice

Mice deficient in the *Kcnj13* gene were generated using the Velocigene method [19] in the C57Bl/6NTac background (Velocigene allele identification number VG19817). The targeted deletion of *Kcnj13* gene removes the full coding sequence. Mice with gene-targeted disruption of the *Kcnj13* gene encoding Kir7.1 K^+^ channel (*Kcjn13* ^-/-^, *Kcjn13* null mutant mouse), wild-type (WT, *Kcjn13* ^+/+^) and heterozygous (*Kcjn13* ^+/-^) littermates were used for all comparisons. Heterozygous mice were mated to produce *Kcjn13* ^-/-^, WT and heterozygous progeny.

Ages of mice analyzed are given as embryonic day (E) or days post conception (dpc), where the presence of vaginal plugs was considered as embryonic day E0.5 or 0.5 dpc. Timed pregnant females (E15.5 to E18.5) were sacrificed by cervical dislocation. Embryos were dissected from the uteri and placed in PBS. Body weight of embryos or newborn pups (P0) was measured in an analytical balance after drying off excess fluid.

Cages containing pregnant mice were surveyed from 7:00 AM on the computed day of birth (E19.5) and new born pups checked for activity and signs of dehydration. After 2 h the whole litter was removed for study. Pups presenting smaller size, signs of dehydration, failure to suckle or breathing difficult were presumed to be of *Kcjn13* ^-/-^ genotype, and were euthanized first. Mice were individually labelled and decapitated for further study. To obtain new *Kcjn13* ^+/-^ mice, only pups presumed to be of *Kcjn13* ^-/-^ genotype were removed and euthanized. The genotype was confirmed to check for a Mendelian distribution of genotypes.

Tail snip DNA was used for genotyping by PCR using the following 3 primers: MP08 for, located in *Kcnj13* exon 2 (5’ATCTTCCCGCTAACCTAT3’), SD rev located in the 3’ untranslated region of the *kcnj13* gene (5’ CAGCTTTCTACCCAGGGAGC 3’) and Neo for, located in the selection cassette (5’ TCATTCTCAGTATTGTTTTGCC 3’). Mice were maintained on standard laboratory chow (2019S diet, Harlan Laboratories) and water *ad libitum*, under regulated temperature (22–26°C) and light (12:12-h light-dark cycle) conditions. Housing ad breeding was at the SPF animal facility of the Centro de Estudios Científicos (CECs), Valdivia, which is accredited by the Association for Assessment and Accreditation of Laboratory Animal Care. Animal procedures were approved by the CECs Animal Care and Use Committee.

For the analysis of Kir7.1 expression, total RNA was isolated from dissected lung and brain from WT, heterozygous and null mutant mice in TRIzol following the manufacturer’s instructions. The RNA was quantified and tested for quality using spectrophotometry and 1% agarose gel. Five μg of total RNA was reverse transcribed at 42°C for 90 min using random primers and ImProm II kir (Promega) to synthesize single-stranded cDNA. The amplicon of Kir7.1 was obtained by PCR using specific primers: For1Ex3Kir7.1 (5’-ATTCTTCATCTTCCCGCTAACCTA-3’) and RevEx3Kir7.1 (5’-GATTTCCCCAGTGCCTTCTTG-3’). Cyclophilin A is used as housekeeping gene.

Ages of mice analysed are given as embryonic days (E) or days post conception (dpc), where the presence of vaginal plugs was considered as embryonic day E0.5 or 0.5 dpc. Birth took place at E19 (P0). Timed pregnant females (E15.5 to E18.5) were sacrificed by cervical dislocation. Embryos were dissected from the uteri and placed in PBS. Body weight of embryos or newborn pups was measured in an analytical balance after drying off excess fluid.

### Tissue preparation

Whole embryos (E14.5 to E18.5), P0 dissected tissues or adult tissues were fixed in 4% paraformaldehyde in PBS for 90 min at room temperature. Then, tissues were dehydrated in 30% (vol/vol) sucrose solution for a week, and cryoprotected with Optimal Cutting Temperature Compound (OCT, Tissue-Tek, USA). Cryosections (10 μm) were mounted on Superfrost/Plus (Fisherbrand, USA) slides, and stained with hematoxylin and eosin (H&E) for lung morphometry or reserved for detection of β-galactosidase activity. For immunohistochemistry assays, newborn lungs (P0) or adult mice (2 months old) were fixed in 4% paraformaldehyde in PBS (pH 7.4) overnight at 4°C, washed in PBS, and embedded in paraffin.

### β-galactosidase activity

The expression of Kir7.1 channel was observed indirectly through the detection of β-galactosidase activity. OCT preserved tissues were cut into 10 μm sections and fixed with 4% paraformaldehyde for 10 min at room temperature, rinsed with PBS and incubated in X-Gal staining solution (PBS pH 7.4, containing 1 mg/ml X-Gal, 2.5 mM K_3_Fe(CN)_6_, 4 mM K_4_Fe(CN)_6_, 1.2 mM MgCl_2_, 0.01% sodium deoxycholate and 0.02% Igepal) overnight at 37°C. Sections were washed with PBS and then counterstained with eosin. Digital images were collected using an Olympus CX31 microscope and recorded with a digital microscope camera (Mshot MD90).

### Immunohistochemistry

Sections (4 μm) were treated with 10% hydrogen peroxide, blocked with 2.5% normal horse serum, and incubated with anti-Kir7.1 [[5], 1:15,000] or anti-pro-SPC (1: 2,000; Millipore) overnight at 4°C. Detection of antigen/antibody complexes was performed with ImmPRESSTM (Peroxidase) Polymer Anti-Rabbit Ig Reagent (Vector Laboratories, USA). The reaction was visualized using Vector SG Peroxidase (HRP) Substrate Kit (Vector Laboratories, USA). In controls the incubation with primary antibody was omitted. Sections were counterstained with Vector Nuclear Fast Red (Vector Laboratories, USA).

### Lung morphometry

Ten random representative fields per H&E stained sample were acquired with the Mshot MD90 camera and Olympus CX31 microscope. All images at a magnification of 400X were taken under the same exposure settings. The percentage of terminal sac spaces is the proportion of white surface area of each field relative to the total image area.

### Cartilage and skeletal staining

Newborn mice were sacrificed by intraperitoneal injection of anesthetic and the skin and internal organs were carefully removed. The remaining carcass was fixed in 100% ethanol for 24 hours. Fat was removed with acetone overnight. Staining was carried out with Alcian Blue (0.3% Alcian Blue 8GS) and 0.1% Alizarin Red for 3–4 days at 37°C on a rocker. The tissues were washed with deionized H_2_O and then 1% KOH for 24 h. Clearing was carried out by consecutive changes of glycerol (20%, 50%, 80%) in 1% KOH every 24 h and the embryos were stored in 80% glycerol/1% KOH. Littermates were used and the results were obtained from at least three mice per genotype.

## Results

**Fig 1:**
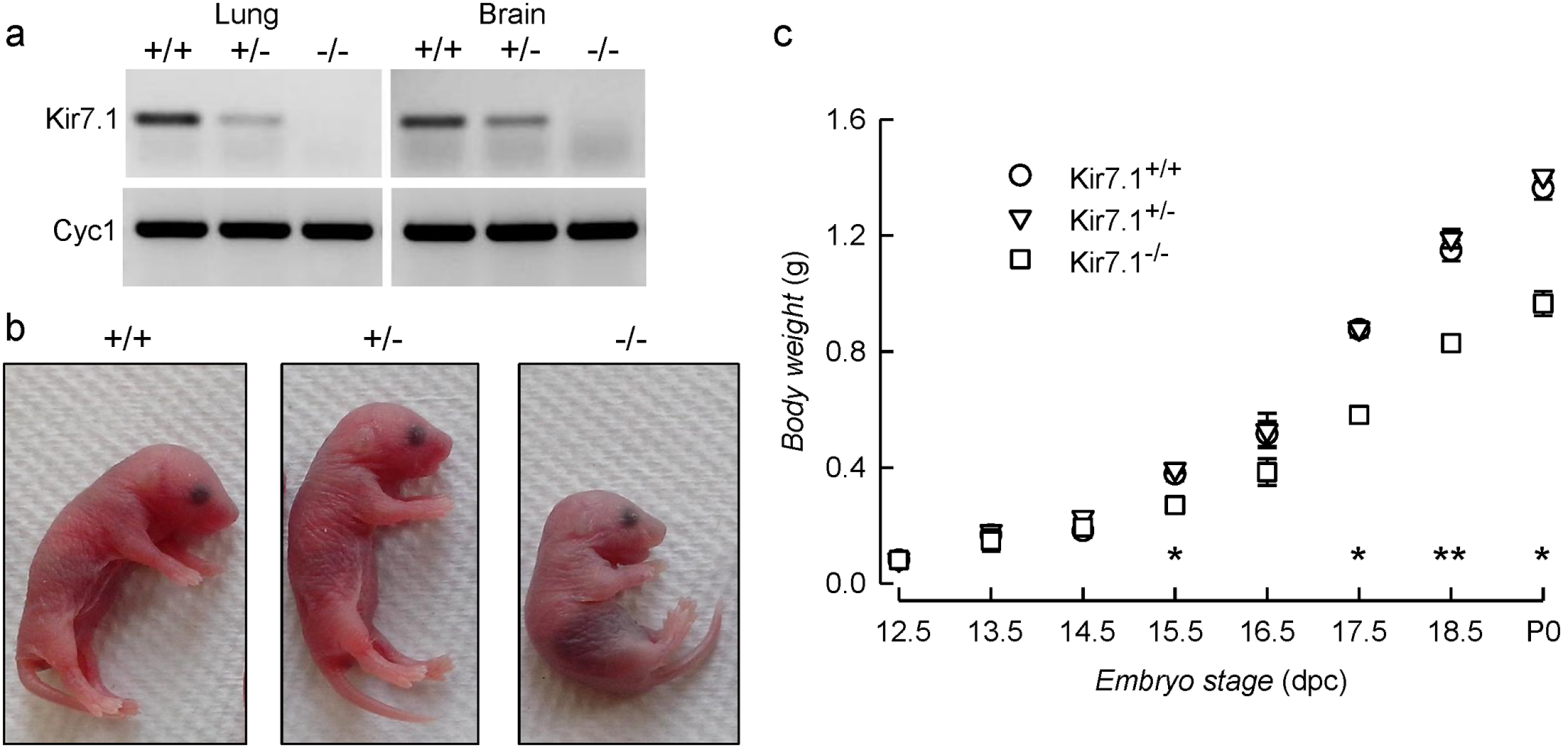
Morphology and body weights of *Kcnj13* null mutant mice. a. Analysis of Kir7.1 expression in WT, heterozygous and null mutant mice; cyclophilin A (Cyc1) is used as constitutively expressed control gene. b. Gross morphology of WT, and heterozygous and homozygous *Kcnj13* null mutant newborn pups. c. Body weight vs. embryonic stage for WT (circles), *Kcnj13* ^*+/-*^ (triangles), and *Kcnj13* ^-/-^ (squares) embryos. Results are expressed as mean ± S.E.M. of the following numbers of embryos: 12.5 dpc: WT 3, *Kcnj13* ^*+/-*^ 9, *Kcnj13* ^-/-^ 4; 13.5 dpc: WT 9, *Kcnj13* ^*+/-*^ 14, *Kcnj13* ^-/-^ 2; n 14.5 dpc: WT 3, *Kcnj13* ^*+/-*^ 11, *Kcnj13* ^-/-^ 6; 15.5 dpc: WT 7, *Kcnj13* ^*+/-*^ 20, *Kcnj13* ^-/-^ 14; 16.5 dpc: WT 7, *Kcnj13* ^*+/-*^ 5, *Kcnj13* ^-/-^ 4; 17.5 dpc:WT 5, *Kcnj13* ^*+/-*^ 12, *Kcnj13* ^-/-^ 6; 18.5 dpc: WT 4, *Kcnj13* ^*+/-*^ 5, *Kcnj13* ^-/-^ 6; P0: WT 7, *Kcnj13* ^*+/-*^ 15, *Kcnj13* ^-/-^ 10. * p< 0.001; ** p <0.05 for the differences between *Kcnj13* ^-/-^ and *Kcnj13* ^*+/+*^ data (ANOVA).

In order to study the physiological role of Kir7.1 inwardly rectifying K^+^ channel we generated Kir7.1 deficient mice by ablation of its codifying *Kcnj13* gene by the Velocigene method [19]. Effective gene deletion was evaluated by studying the gene expression in lung and brain tissue of P0 pups by RT-PCR (Fig 1a). The transcript was absent from *Kcnj13* ^-/-^ mutant mice as expected. Heterozygous *Kcnj13* ^+/-^ mice were indistinguishable from their wild type *Kcnj13* ^+/+^ litter mates in growth and development. Homozygous mutant mice however failed to suckle, were often cannibalised by their mothers and did not survive beyond P0. Because of extensive cannibalism we were initially under the impression most of the null mice died *in utero*, but cesarean delivery and genotyping revealed a Mendelian distribution of the three genotypes in embryos generated after crossing heterozygous mice (Table 1). Also, careful surveillance and separation of newborn mice showed that delivery produced litters with Mendelian distribution of genotypes (Table 1). Newborn null mutant mice were slightly smaller than their heterozygous of wild-type littermates (Fig 1b). This difference was significant and was also present in the embryos from stage E15.5 onwards (Fig 1c).

**Table 1:** Survival of *Kcnj13* ^-^ mutant mice.

	*Kcnj13* ^-/+^	*Kcnj13* ^+/+^	*Kcnj13* ^-/-^	Total	*χ* ^*2*^ 2df	*P*
**E11.5–18.5**	53 (56)	122 (112)	51 (56)	224	1.5	0.9
**P0**	16 (16)	33 (32)	15 (16)	64	0.09	0.98

**Fig 2:**
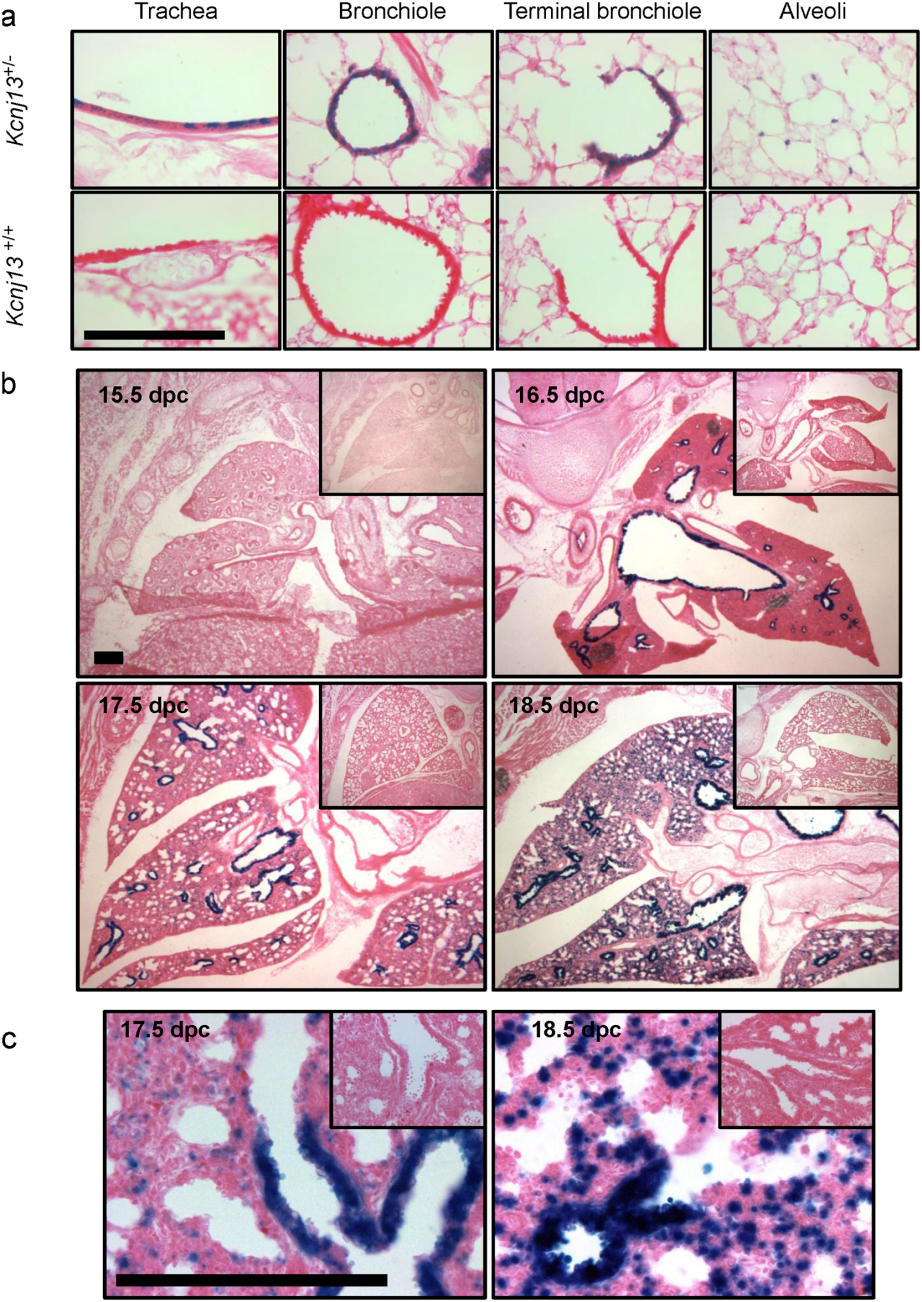
Sites of Kir7.1 channel expression in the respiratory system. a. Tissue cryostat sections (10μm) from trachea, bronchiole, terminal bronchiole and alveoli) of two month old *Kcnj13* ^+/-^ (top) and *Kcnj13* ^+/+^ (bottom) mice were stained with X-Gal (blue) and counterstained with eosin. b. Kir7.1 channel expression during respiratory system development. Tissue cryostat sections were stained with X-Gal and counterstained with eosin. The embryonic age is indicated. c. Higher magnification views of tissues at embryonic stages E17.5 and E18.5. Bars represent 100 μm.

**Fig 3:**
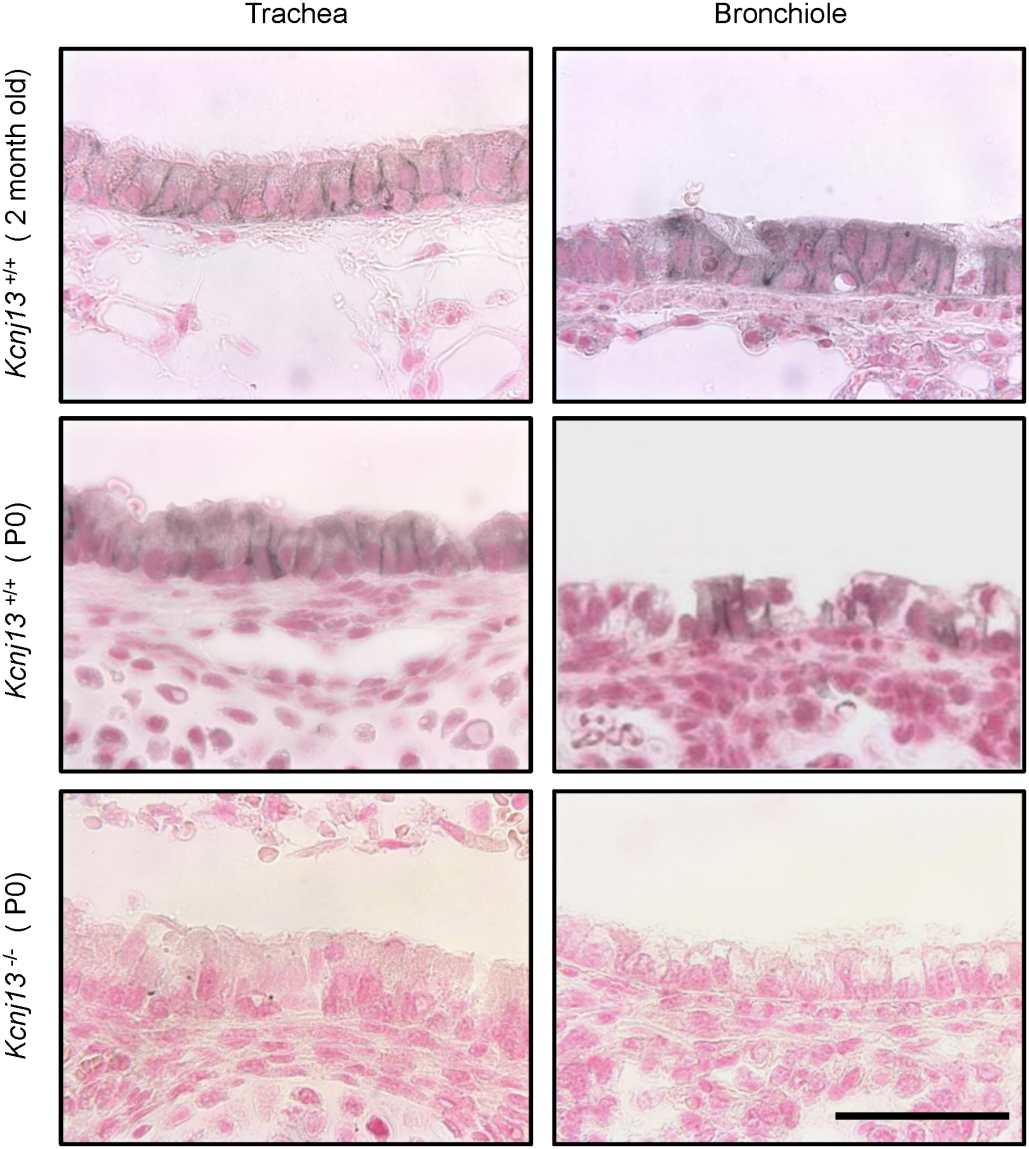
Basolateral expression of Kir7.1 channel in the epithelium of the airways. Immunohistochemical detection of Kir7.1 channel in trachea (left) and bronchiole (right) in adult *Kcnj13* ^+/+^, newborn *Kcnj13* ^+/+^ or newborn *Kcnj13* ^-/-^ mice. Tissue sections were treated with anti-Kir7.1 antibody (1:15,000). Kir7.1 expression was restricted to the basolateral membrane of airway epithelium in adult and newborn *Kcnj13* ^+/+^ mice. Staining in *Kcnj13* ^-/-^ tissues shows complete absence of specific immunoreactive signal. Nuclei were counterstained with Fast Red. Scale bar represents 50 μm.

Respiratory distress consequent to delayed lung development is a frequent cause of perinatal mortality. There are no reports of Kir7.1 expression in the respiratory system of the mouse or any other species. We have used the expression of the bacterial β-galactosidase (LacZ) reporter that is driven by the *Kcjn13* promoter in tissues from *Kcjn13* ^+/-^ and *Kcjn13* ^-/-^ mice to check for expression in the channel in the respiratory system. Kir7.1 was expressed in mouse airways and lungs as seen from the presence of LacZ activity (Fig 2a) in the epithelium of the trachea, bronchioles and terminal bronchioles as well as in the alveoli of *Kcjn13* ^+/-^ mice. In this last location the stain appeared to associate with type II pneumocytes (not shown). Analysis of embryonic tissue (Fig 2b and 2c) revealed that LacZ, and therefore Kir7.1 channel expression was absent at 15.5 dpc but emerged at 16.5 dpc and was present at delivery. Higher magnification showing the epithelial expression of Kir7.1 is presented in Fig 2c. The presence of Kir7.1 protein in the respiratory system was corroborated by immunolocalization. The immunolabel was present in trachea, bronchi and lung tissue of P0 and adult mouse, in the first two tissues at the basolateral aspect of epithelia cells (Fig 3).

**Fig 4:**
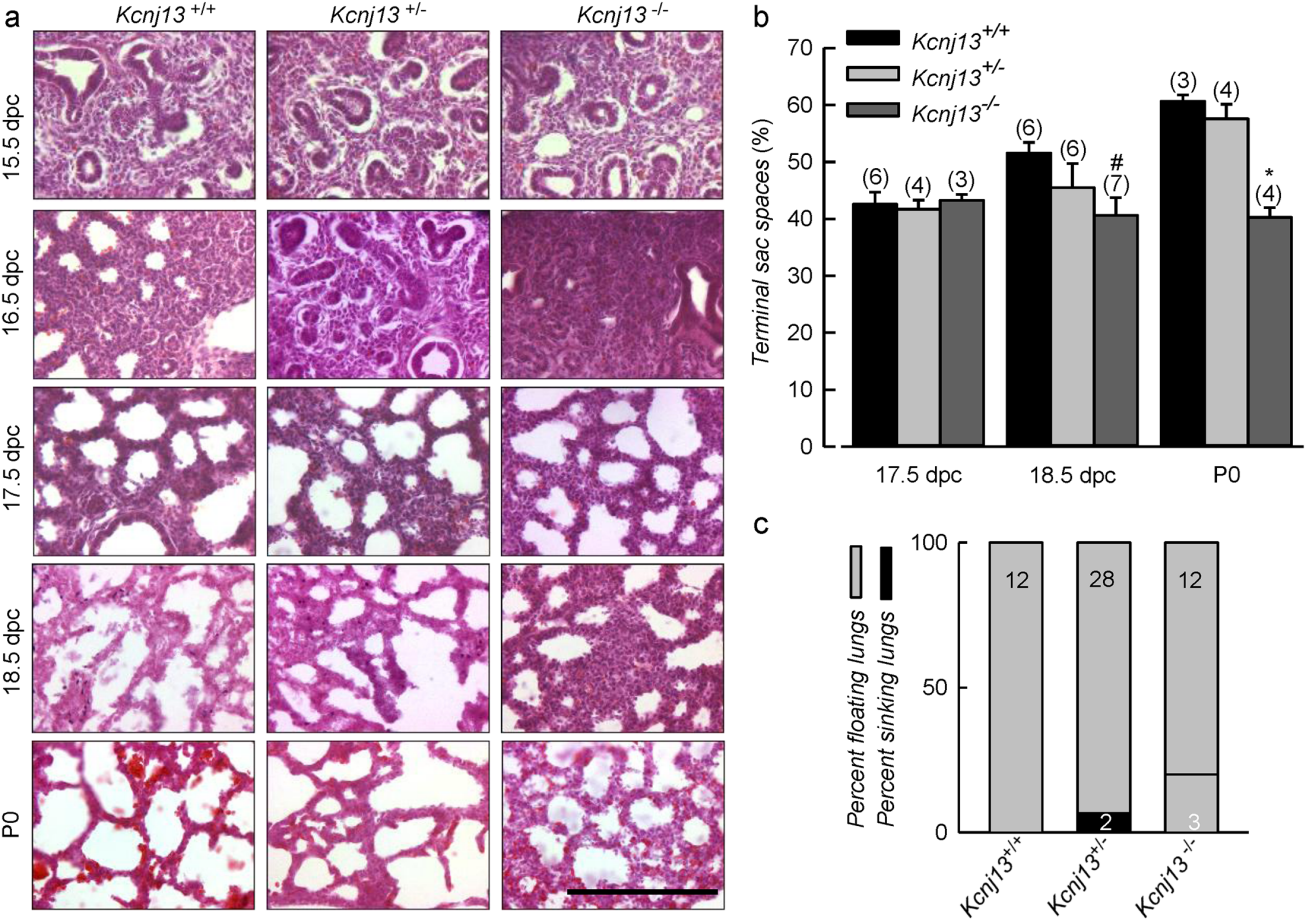
Pulmonary abnormalities in embryonic lungs from *Kcnj13* ^-/-^ mice. a. Hematoxylin and eosin stained lung sections taken at various gestational stages as indicated. Morphological differences in KO lungs were observed at E18.5 and P0. Null mutant mice show a lower air space and thicker walls at lung terminal sacs compared to WT and heterozygous mice. No differences were visible between *Kcnj13* ^+/+^ and *Kcnj13* ^+/-^ genotypes. Scale bars represent 100 μm. b. Morphometric analysis of terminal sac spaces in lungs at various gestational stages. Significant reduction in spaces was observed in Kir7.1 deficient mice from E18.5 onwards. Results are expressed as mean ± S.E.M, # p<0.05 and * p<0.01 for the difference with WT by ANOVA. c. Graphical representation of newborn lung flotation test. Grey sections of columns correspond to percent of floating lungs, with black being the percent sinking lungs.

Does the absence of Kir7.1 channel from null mutant mice have any effect in lung development? This was examined histologically as seen in Fig 4a, that shows tissues from mice of the three genotypes in embryos from 15.5 to 18.5 dpc. No major differences between genotypes can be seen up to 17.5 dpc. At E18.5 and P0, when embryonic mouse lung develops from canalicular stage to saccular stage, there appears that spaces in the *Kcjn13* ^-/-^ tissue are smaller than in the WT or heterozygous tissue. This impression is borne out by the quantification of terminal sac spaces shown in Fig 4b. There was no difference between genotypes at 17.5 dpc, but terminal sacs of Kir7.1 deficient mice were significantly smaller than those in WT at 18.5 dpc, and that those of WT and heterozygous mice at P0. Failure to inflate lungs is not an uncommon consequence of lung immaturity and is encountered in several knockout mouse models [20]. *Kcjn13* ^-/-^ mice survived for up to 12 h after birth and did not appear cyanotic, suggesting an absence of respiratory distress. This is consistent with similar levels of expression of proSp-C (surfactant protein C) in lungs of *Kcnj13* ^+/+^ and *Kcnj13* ^-/-^ P0 mice (not shown). Lungs dissected from *Kcjn13* ^+/+^, *Kcjn13* ^+/-^ and *Kcjn13* ^-/-^ P0 mice floated when placed in saline solution suggesting that normal respiration had taken place (Fig 4c). Although some sinking lungs cropped up in the null mutant and heterozygous mouse groups this tendency did not reach statistical significance.

**Fig 5:**
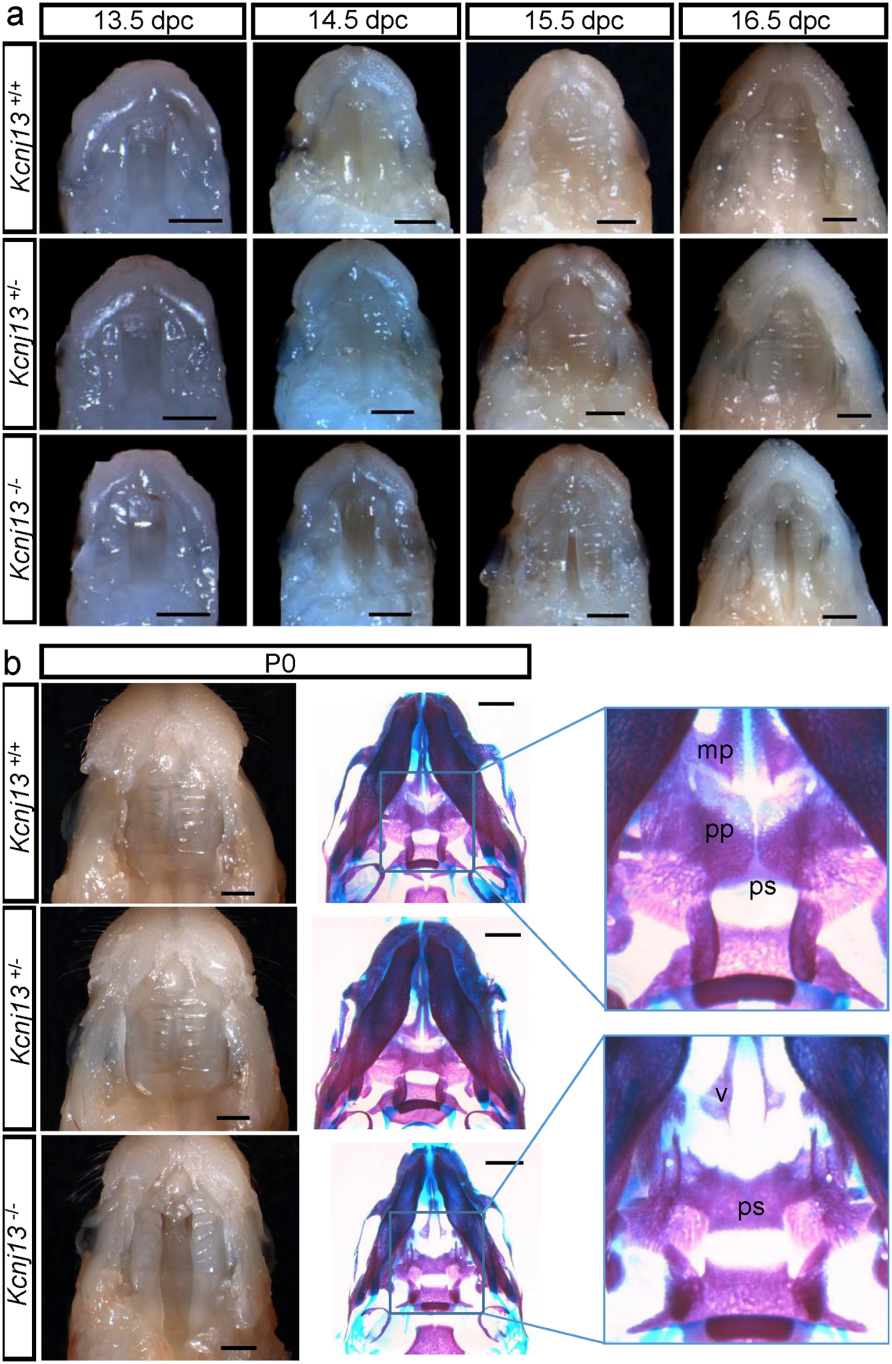
Palate formation during embryonic development in *Kcnj13* ^+/+^, *Kcnj13* ^+/-^ and *Kcnj13* ^-/-^ mice. a. Dissected midfacial segments (without brain, mandible and tongue) from mouse heads collected from embryos 13.5–16.5 dpc. b. Mouse heads on postnatal day (P0) with dissected midfacial segments (left) and alizarin red and alcian blue skeletal staining (right). The palatine (pp) and maxillary (mp) processes are indicated In the *Kcnj13* ^-/-^ specimen both the presphenoid (ps) and the vomer (v) are visible. Bar, 1 mm.

Another cause of early postnatal lethality in null mouse models is a developmental craniofacial alteration leading to cleft palate [20] and a recent study of mouse palatal transcriptome suggests *Kcnj13* gene as a potential key regulator of palatal development [21]. *Kcjn13* ^-/-^ mice do indeed show a deficiency in the process of palate fusion during development. Palate formation in mice takes place by fusion of so called palatal shelves that are paired outgrowths initially growing vertically flanking the developing tongue (~13.5 dpc) to then progressing horizontally towards the midline above the tongue (~14.5 dpc). Fusion of the palatal processes has already occurred by dpc 15.5 [22]. Fig 5a shows macroscopic views of the roof of the mouth of *Kcjn13* ^+/+^, *Kcjn13* ^+/-^ and *Kcjn13* ^-/-^ mice. Palatal processes that are evident at 13.5 and 14.5 dpc in WT and heterozygous embryos have disappeared at 15.5 dpc, at which time complete sealing of the palate is evident. This was not the case for *Kcjn13* ^-/-^ embryos that show a delayed horizontal growth from E14.5 and lack of fusion at 15.5 and 16.5 dpc. Fig 5b shows the status of palate fusion in *Kcjn13* ^+/+^, *Kcjn13* ^+/-^ and *Kcjn13* ^-/-^ newborn mice, with the latter presenting evident cleft palate. Also shown are preparations to reveal bone and cartilage (blue), where it can be seen that palatine (pp) and maxillary processes (mp) are extended to the midline in *Kcjn13* ^+/+^ and *Kcjn13* ^+/-^ tissues. In the *Kcjn13* ^-/-^ mouse, these processes were absent thus exposing the vomer (v) and presphenoid (ps) bones to result in a complete cleft secondary palate.

**Fig 6:**
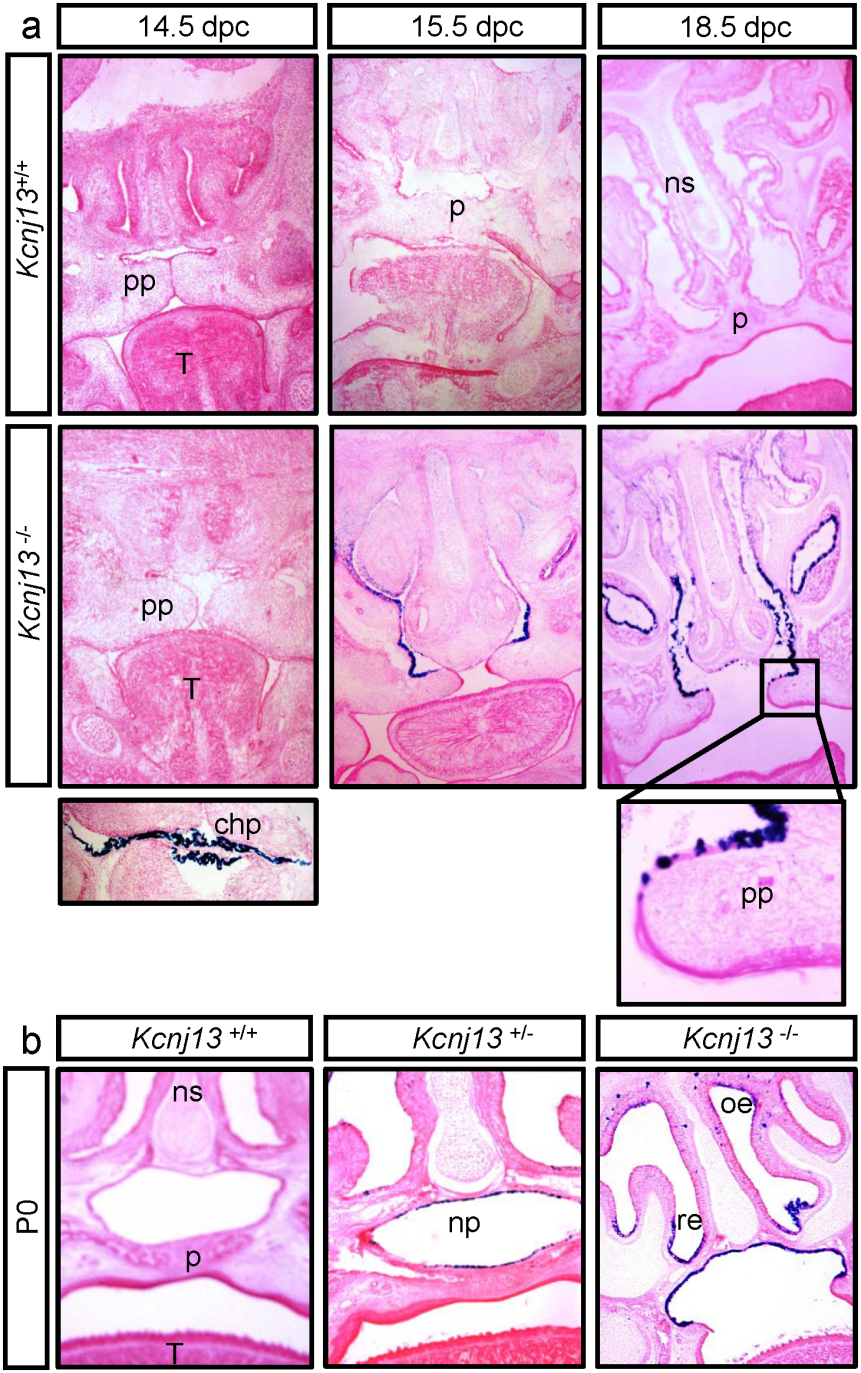
Expression of Kir7.1 in the developing palate of the mouse. Coronal sections of embryo heads at E14.5, E15.5 and E18.5 (a) or postnatal day P0 (b). *Kcnj13* ^+/-^ and *Kcnj13* ^-/-^ samples show β-galactosidase staining (dark blue) in cells that express Kir7.1. a. β-galactosidase activity can be observed at E15.5 mainly in respiratory epithelium and at the palatine process (pp) at E18.5 (enlarged view in inset). The choroid plexus (chp) is shown as positive control at E14.5. b. β-galactosidase activity in mice at P0 is observed in palatal (pe) (nasal side), respiratory (re) and olfactory epithelium (oe). Each section was photographed at 40X magnification. ns: nasal septum, p: palate, T: tongue.

We undertook a histological examination of the developing palate in *Kcjn13* ^+/+^ and *Kcjn13* ^-/-^ embryos. Fig 6a shows medial coronal sections that reveal the palatal processes apposed but not fused at E14.5 in both *Kcjn13* ^+/+^ and *Kcjn13* ^-/-^, and already fused at E15.5 in the WT but not in the null mutant mouse. The sites of Ki7.1 expression were identified by β-galactosidase staining of *Kcjn13* ^-/-^ mice. Kir7.1 expression is absent at 14.5 dpc, a stage at which robust expression is already present in the choroid plexus (see inset). Kir 7.1 expression is evident at E15.5 in the respiratory epithelium as well as that covering the palatal processes but here only in the nasal side. The same is seen at E18.5, where the higher magnification picture corroborates that Kir7.1 expression stops towards the tip of the palatal process. Fig 6b shows that Kir7.1 expression persists after birth and covers the epithelium of the nasopharinx in the non-cleft palate *Kcjn13* ^+/-^ tissue. Expression is also present in the respiratory and olphactory epithelia after birth as seen in the *Kcjn13* ^-/-^ section.

## Discussion

*Kcjn13* null mutant mice suffer from early postnatal mortality with pups not surviving further than P0. Our initial impression that *Kcjn13* ^-/-^ mice might not experience normal *in utero* development were dispelled by examining the distribution of phenotypes of 11.5–18.5 dpc foetuses of pregnant *Kcjn13* ^+/-^ females that had been crossed with heterozygous *Kcjn13* ^+/-^ males. The distribution of genotypes was not significantly different from the expected Mendelian distribution of 25% *Kcjn13* ^+/-^/50% *Kcjn13* ^+/+^/25% *Kcjn13* ^-/-^. The process of generation of *Kcjn13* ^-/-^ mouse results in the insertion of a DNA coding a β-galactosidase whose activity is now under the control of the *Kcjn13* promoter and therefore reports the channel tissue distribution. Sites of expression of Kir7.1 thus identified included the brain, meninges and choroid plexus; in the eye the retinal pigment epithelium; the gall bladder, and the cricoid cartilage; the small intestine and the thyroid gland. Most of these locations have been identified before as sites of expression of Kir7.1 [11].

Spurred by the frequent occurrence of respiratory distress as a cause of perinatal lethality, we looked for Kir7.1 expression in the respiratory system. There is extensive expression of the channel in the respiratory tree, including the epithelia of the trachea, bronchioles and alveoli, where Kir7.1 appears to be associated with type II pneumocytes. Expression of Kir7.1 in the respiratory system arises at E16.5. There was some differences in the developing lung between *Kcnj13* null mutants and heterozygous and WT mice, with the terminal sac spaces in tissue from the *Kcnj13* ^-/-^ mice significantly smaller from E18.5 through to P0. We do not think, however, that this difference can lead to a lethal phenotype as the surviving P0 mice were able of breathing and were not cyanotic, and lungs had inflated as attested by flotation tests.

What might be the reason for this retardation at the saccular development phase or indeed the function of the Kir7.1 channels in adult respiratory epithelium? It is well known that the transport of ions and fluid across all parts of the respiratory system is vital for gas exchange function. Mucociliary clearance is a key element in normal lung innate immune system function. Clearing the airways of particles including pathogenic organisms occurs through ciliary beat and ion transport and its associated water flow into and from the fluid layer covering all portions of the respiratory tract. The electrolyte transport systems that control the airway fluid layer include absorptive as well as secretory functions. Absorption requires the presence of apically located epithelial Na^+^ channel ENaC whilst secretion relies on Cl^-^ channels such as CFTR or Ca^2+^-activated TMEM-16 Cl^-^ channels. In both cases the activity of basolateral Na^+^/K^+^ pump is also essential, as is the presence of K^+^ channels required for K^+^ recycling and to maintain a negative membrane potential compatible with continued transport of Na^+^ or Cl^-^ [23,24]. Kir7.1 could fulfill this role in the cells of the respiratory epithelium where it is expressed, but it must be pointed out that several other K^+^ channels have also been identified in the alveolar and airway epithelia [25] suggesting a degree of functional redundancy.

As to the retardation in saccular expansion noticed in *Kcnj13* ^-/-^ mice we can only conjecture that this might be due to a requirement of the Kir7.1 channel in some aspect of the process of expansion of the intra-sac spaces. Fluid secretion is thought to play a role in lumen expansion in vertebrate organogenesis driving expansion of nascent lumens to form a single tube or a sac with CFTR and Na^+^/K^+^-pump being often central drivers of the process [26]. There is early evidence that this concept might apply to lung morphogenesis as suggested by its dependence upon adequately regulated intra-lung hydrostatic pressure whose modulation affects organ growth and the development of alveoli [27]. It is tempting to speculate that a deficit in fluid secretion due to the absence of Kir7.1 might be responsible for retarded saccular expansion in *Kcnj13* ^-/-^ mice.

K^+^ channels have been proposed to fulfil diverse functions in lung and airway physiology and unravelling the precise role of Kir7.1 will be a daunting task. In a recent review that summarises the evidence for the presence of more than 40 transcripts for K^+^ channels in airway and alveolar epithelium, Bardou et al. {Bardou, 2009 7315 /id} use the following understatement: “The physiological and functional significance of this high molecular diversity of lung epithelial K^+^ channels is intriguing”. This diversity includes members of all major classes of K^+^ channels: voltage-dependent Kv channels, leak two-pore domain K_2P_ and inwardly rectifying Kir channels, and functions as diverse as gas exchange and alveolar stability, inflammatory responses, epithelial repair after injury, control of transepithelial fluid and ion transport, Cl^-^ secretion and Na^+^ absorption. Kir7.1 might contribute to one or more of these functions, perhaps in conjunction with other K^+^ channels. Discovering its contribution to lung and airway physiology will call for more tissue/cell-directed animal modification to allow the study of Kir7.1 avoiding the lethality of the total gene inactivation presented here.

Examination of head morphology suggested some sort of subtle craneo-facial malformation in *Kcnj13* ^-/-^ mice. Detailed observation revealed that these mice suffered from complete secondary cleft palate, which is the most probable cause of their early postnatal death [20]. Palatogenesis in mammals is a precisely orchestrated tissue growth and reorganisation in which so-called palatal shelves grow from the medial nasal and maxillary prominences to fuse together thus creating a vault at the top of the oral cavity. Our histological examination of palate formation shows that normal initial elevation of the processes and horizontal growth of the palatal shelves does occur as expected for both WT and *Kcnj13* null mutant embryos. Despite the fact that processes do make midline contact at 14.5 dpc in *Kcnj13* ^-/-^ embryos, fusion has not occurred at 15.5 dpc as it does in WT embryos.

Cleft palate is one of the most common birth defects in humans, it includes syndromic and nonsyndromic forms and is influenced by genetic and environmental factors [28–30]. A growing number of molecules has been related with this malformation, mainly extracellular signalling and transcription factors and to a lesser extent cell adhesion molecules and extracellular matrix, cholesterol pathway and kinase cascade components [31]. Most of the genes involved in secondary cleft palate in humans have also been implicated in mouse models of the disorder, with important proposed roles in palate fusion for transforming growth factor-β 3 (TGF-β 3), Smad and bone morphogenetic protein (BMP) proteins [21,32]. The relevance of ion channels has been less well explored.

Mice deficient in the inwardly rectifying Kir2.1 channel die postnatally as a consequence of a complete cleft of the secondary palate [33]. Interestingly null mutant mice for TGF-β 3 die soon after birth exhibiting delayed pulmonary development and cleft palate [34,35]. It is thought that TGF-β 3 could play an important role in palate fusion through a central role in the signalling pathway controlling a process that may involve epithelial-mesenchimal transition, apoptotic cell death or cell migration [22]. Mutations the human Kir2.1 channel have been shown to be responsible for the craniofacial and digital defects of Andersen-Tawil syndrome. Kir2.1 knockout mice also show digital defects in addition to cleft palate and these defects appear strikingly similar to those that occur upon interference with TGF β/BMP, Wnt-Wingless (Wg) or Notch signalling [36]. Using *Drosophila* as a model, these authors show that the fly homologue of Kir2.1 affects development perhaps by interfering with *Drosophila* decapentaplegic (Dpp) signalling. As Dpp is considered analogous mammalian BMP, they propose that an effect of Kir2.1 activity on the BMP pathway could be responsible for the morphological defects in the Kir2.1 deficient mouse.

The expression of Kir7.1 coincides with the moment of palatal process fusion, i.e. between dpc 14.5 and 15.5. Interestingly it appears that the channel is expressed by epithelial cells covering the dorsal aspect only of the palatal shelves and it might be absent from the point of contact between opposite between processes itself. This contrasts with the localization of TGF-β 3 that is present in the MEE edge and the tip epithelium of the nasal septum [37]. It is clear that the presence of Kir7.1 is essential for the process of fusion of palatal processes during embryonic development and it is possible that its activity as a channel, controlling the membrane potential or mediating transmembrane K^+^ fluxes in the palatal shelf epithelium, might be a triggering element in palate sealing. It will be interesting to explore further this possibility perhaps in rescue experiments *in vitro*. Effects of K^+^ channels not involving their permeation properties have also been seen to play a role in cancer cell migration [38] and this type of mechanism, that probably involves interactions with other integral membrane proteins but is as yet poorly understood, could be important in the context of Kir7.1 function in palatogenesis. More generally, K^+^ channels have often been invoked as central participants in cell migration, proliferation and differentiation processes [39], and any one or more of these could underpin the role of Kir7.1 in palatal sealing event.

Although we lack mechanistic explanations for the effect of Kir7.1 inactivation on palatal formation and on lung development, we provide firm evidence of its involvement in these processes. We hope that this evidence will spur new work that might contribute to our understanding of these important biological processes.
